# CATSWoTS: Context Aware Trustworthy Social Web of Things System

**DOI:** 10.3390/s19143076

**Published:** 2019-07-12

**Authors:** Sabeen Javaid, Hammad Afzal, Fahim Arif, Naima Iltaf, Haider Abbas, Waseem Iqbal

**Affiliations:** Department of Computer Software Engineering, National University of Sciences and Technology (NUST), Islamabad 44000, Pakistan

**Keywords:** context-aware, Internet of Things, IOT, quality of service, SOA, trust management, WoT

## Abstract

The inevitable revolution of the Internet of Things (IoT) and its benefits can be witnessed everywhere. Two major issues related to IoT are the interoperability and the identification of trustworthy things. The proposed Context-Aware Trustworthy Social Web of Things System (CATSWoTS) addresses the interoperability issue by incorporating web technologies including Service Oriented Architecture where each thing plays the role of a service provider as well as a role of service consumer. The aspect of social web helps in getting recommendations from social relations. It was identified that the context dependency of trust along with Quality of Service (QoS) criteria, for identifying and recommending trustworthy Web of Things (WoT), require more attention. For this purpose, the parameters of context awareness and the constraints of QoS are considered. The research focuses on the idea of a user-centric system where the profiles of each thing (level of trustworthiness) are being maintained at a centralized level and at a distributed level as well. The CATSWoTS evaluates service providers based on the mentioned parameters and the constraints and then identifies a suitable service provider. For this, a rule-based collaborative filtering approach is used. The efficacy of CATSWoTS is evaluated with a specifically designed environment using a real QoS data set. The results showed that the proposed novel technique fills the gap present in the state of the art. It performed well by dynamically identifying and recommending trustworthy services as per the requirements of a service seeker.

## 1. Introduction

The unfolding of the Internet of Things (IoT) revolution has taken place so rapidly before our eyes that it has started dominating the different spheres of life. From smart health to smart home, from smart city to smart community, and smart applications for businesses, the services of IoT are playing a significant role. The all-encompassing term of IoT is a network that connects the physical world with the cyber world through smarts objects, sensors, actuators, Radio Frequency Identification (RFID) tags and even through virtual objects and things [1]. The future of IoT seems to be very promising.

IoT connects billions of heterogeneous physical and virtual objects. The issue of interoperability is attempted to resolve by exploiting the concept of web technologies hence devising the term of Web of Things (WoT). WoT extends the concept of IoT. WoT connects devices to the Internet, incorporate web servers, models the services and then introduces these services as RESTful [2]. Web services are being used to fully integrate distributed and heterogeneous devices. The web services are the foundational elements of Service Oriented Architecture (SOA). In SOA systems, devices can play the role of a service provider and service consumer as well if needed. The compatible service APIs make usages of SOA technologies like REST, WS-* and CoRE [3]. An Arrowhead framework [4] based on SOA for enabling the interoperability among billions of heterogeneous devices is also proposed. In short, WoT lets the real-world and virtual objects be part of the World Wide Web (WWW). This is the reason that the concept of WoT is incorporated in the proposed research to resolve the interoperability issue amicably.

Social IoT encompasses social networks and peer-to-peer (P2P) networks in which autonomous social relations can be established [1]. The IoT gets social when things become smarter. In this kind of network, things may provide services and things may seek services from others. Besides this, recommendations can be taken for trustworthy things including the physical and cyber world. The things are owned by humans and worked for humans; therefore, it is important to consider the social relations among the owners of the things [1]. Keeping in view that the importance of social relations for humans and the trust level they put on these relations is more than they put on strangers; this important aspect is considered in this research.

In the presence of billions of service providers, finding trustworthy services is not an easy task. Evaluating the trustworthiness of a service provider is a critical task. Identifying which parameters are significant, for shortlisting the qualified trustworthy service providers, is a matter of grave concern. In this paper, the problem of finding a trustworthy service provider has been tackled through proposing a trust recommender and then managing the trust for the evaluation of services.

The motivation behind this research is to focus on finding and separating those trustworthy services which not only fulfill the Quality of Service (QoS) criteria like reliability, availability but also the context-awareness. Context is a piece of information that manifests the situation or position of an entity [5], whereas context awareness depicts such a system which provides relevant information based on its context [5]. Though many systems had been proposed for the past two decades; however, with the introduction of IoT, where billions of things connect, context awareness has become a challenge [5]. Collecting and processing data from these things is not an easy task. The data will be valuable only if it has been processed and analyzed. Context-aware computing is significant for evaluating the trustworthiness; therefore, in the CATSWoTS, this aspect is also being considered so that a service seeker may get a list of qualified services based on QoS. Various QoS constraints are considered in the state of the art, but, in this research, three constraints like reliability, availability, and latency (because of their significance) are considered for the evaluation. For context evaluation; location, time and capabilities of a service provider are considered. In short, the main idea is to propose a system that helps in identification of quality IoT services by exploiting the concept of Social Web of Things and then maintains the trust level of such services. The trustworthiness is evaluated through direct experience of a service consumer and indirect experience (also called recommendation).

The contributions of this research are described below:

1. The CATSWoTS is an encouraging step towards the settlement of trustworthy recommendations of IoT services provided either by a social circle or by a third party. The system also considers the self-experience and the recommendation of a centralized level.The authentication and authorization mechanism makes the system security as it limits the access to trusted contacts only.
2. To evaluate and measure the trustworthiness of service, a rule-based collaborative filtering technique is proposed by identifying and integrating the various criteria of context and QoS in Social Web of Things. After recommending a service, feedback is also collected from a service consumer to update the trustworthy level of the consumed service which is then stored to help other service seekers.
3. The experiments are conducted on a real dataset of 2507 services to evaluate the performance of the CATSWoTS. The results show that the proposed technique recommends trustworthy services by fulfilling the criteria of QoS and context awareness of a service seeker with great accuracy.

The remaining of the paper is organized into four sections. Section 2 describes the foundational concepts and state of the art in the related areas. The CATSWoTS is presented in Section 3. In Section 4, the effectiveness of CATSWoTS is demonstrated, whereas Section 5 concludes the research and describes future work.

## 2. Background Study

In this section, the foundational concepts and related work in the area of trust management for IoT and WoT are discussed.

### 2.1. Preliminary Concepts

One of the main ideas from which IoT evolved is Pervasive Computing. “Internet of Things” is the term behind the concept of making connections through networks. IoT is a paradigm which intends sensors, smart objects, and actuators to form a global network that can be uniquely identified based on standard communication protocols. It could be said that it is one paradigm having many visions including things-oriented and internet-oriented attributes [6]. IoT has great potential and is considered as one of the most important key areas for future technology [6]. Few examples of the applicability of IoT can be witnessed in safety protection, surveillance, logistics, and transportation, disaster management, etc. [6].

An architecture for Social IoT was proposed by Atzori et al. [7]. The social relationship between the connected objects was identified. An integral component of this paradigm was Middleware (MW). Middleware architecture following the principles of SOA was proposed [8]. Web of things refers to the integration of web technologies with smart things including services provided by them. The architectural style Representational State Transfer (REST) was used for this purpose [9]. It made services abstract for the selection of best interface without going into technical details. REST has become popular among researchers and is their foremost choice for establishing a global architecture for smart things [1]. The era of the social object is the next phase of IoT [10]. A comparison was made between human and IoT revolution. It was described that a basic knowledge environment was translated into actions. Furthermore, they described different phases of IoT composition [10].

An arrowhead framework [4] was proposed to facilitate the operations of interconnected systems. Based on SOA, it proposed three interoperability approaches which were interoperability layer, the translator system, and the translation as a service. In the interoperability layer, semantics and related technology were defined. The translator system consumed a service and then provided it on another technology, whereas the translation as a service had an extra service that could be consumed for translation purposes. In addition, it had core systems such as service registry, authorization, and orchestration which could be used by any other system. A service or a system could be easily designed and developed with the support of the arrowhead framework. Use cases were designed to evaluate the framework.

Androcec et al. [11] reviewed the usage of semantics for facilitating interoperability in IoT. A comprehensive background study was carried out to give the answers to five major research questions. After that, data was extracted from 105 chosen studies. These scientific works were selected based on six criteria. It was identified that all studies except four discussed OWL (Web Ontology Language). Semantic Sensor Network (SSN) ontology was identified as directly related to IoT. The research concluded that the research area had become mature and ontologies were declared as standards for semantic descriptions. Linked data was projected as an emerging topic and multiple themes were found to exist. In short, it was a comprehensive review of the semantics in IoT. Another approach that addresses the semantic data integration, using the cloud based data as a service (DaaS) layer while ensuring the security of the system is presented in [12].

### 2.2. Trust Management Systems

IoT is a network of interconnected objects and humans as well. The entities can work without the intervention of humans [10]. The presence of IoT devices around humans creates opportunities for malicious activities. Hence, it makes the selection of services among the pool of services a tedious task. Furthermore, access to the data acquired by these entities must ensure the secrecy and privacy of data by emphasizing reputation and trust [13]. A classification tree for trust composition is developed by Guo et al. [14]. In this survey, five design dimensions were identified. The authors describe that, in state-of-the-art approaches, these five dimensions are considered necessary for a trust computation model. It is recognized that the researchers follow one of the sub-dimensions of these five dimensions. Trust composition needs to consider QoS such as reliability, competence, and social relations. Trust evidence needs to be broadcast to peers. It can be done through distributed and centralized approaches. For aggregating trust pieces of evidence, various techniques are identified in this survey which are the weighted sum, belief theory, Bayesian inference, fuzzy logic, and regression analysis. For updating trust, event-driven or time-driven approaches are used. Similarly, single or multi-trust are used for trust formation [14].

A comprehensive review of the state of the art related to IoT architectures, Privacy Enhancing Technologies (PETs), and privacy laws was carried out in [15]. It was identified that implementing PETs at various layers of IoT architecture could ensure the privacy needs. The study elaborated the principles of privacy legislation which were linked with PETs. It was identified that eight basic principles laid the foundation of European privacy laws [15]. Moreover, it was assessed that careful deliberation is required for the usage of PETs.

Sato et al. [16] presented a system for resolving the issue of security and privacy. Different levels were introduced for exchange which was done through a contract. These levels were applied to establish trust between devices and which then monitored and controlled IoT devices. For this, identification and monitoring of devices and connection protocols were suggested. The drawback of this research was that it focused on the privacy and security of data, but the context while trusting on the devices and the trust based on the quality of services were ignored [16].

A trust management protocol [17] was proposed which changed dynamically in IoT. Scalability, adaptability, survivability and the quality at service level were not focused. In another research, Ning et al. proposed a system which focused on data integrity, privacy, and availability [18]. However, trustworthiness in terms of quality of service was not given attention in this research.

Roman et al. [19] focused on managing the authentication, authorization, interoperability, and business model. The challenges faced by distributed computing were focused on this research; however, context-awareness was not given attention in this research. A research work focusing on integrity and confidentiality of real-time IoT systems was done by Kothmayr et al. [20]. A hybrid architecture called DARE based on wireless sensor network and mesh networking was proposed. In [21], an approach for QoS based service selection was proposed. It also considered utility function and distance estimation for the service recommendation in addition to QoS attributes. Pearson Correlation Coefficient (PCC) and the Haversine distance formula were used to improve the performance level. Service categorization was carried out based on distance, PCC, and utility function in order to select the best suitable service. The proposed work only helped in identifying the location and QoS based services and did not consider context related attributes such as capacity and time. In addition, history of service providers considered just for the utility function. No proper mechanism for trust management was presented in this paper.

Saied et al. [22] proposed Context-Aware trust management. After information gathering, an entity was selected and the potential candidate nodes were selected. For the final selection, these selected set of nodes compete, weights were computed and the best-rated nodes were given by the trust manager. After this, a feedback report was sent by the client node to the trust manager which was maintained there for future use. To differentiate it from an adaptive process, a learning phase also called cognitive process was done. The process of updating record was done by the trust manager based on these reports. Through experimental validation, it was identified that several attacks like bad-mouthing, on-off attack were stopped. The research tackled the issue of attack models. However, a QoS service based evaluation was not considered in the research.

Chen et al. [3] introduced a trust protocol for Service Oriented based IoT system. An adaptive filtering technique was devised to regulate the constraints. Validation was done through NS-3 simulation tool. The criteria for indirect (recommendations) experience and direct (experience and feedback) were suggested in a user-centric environment. Three relations like social contact, Community of Interest (CoI) and friendship were considered. A profile was preserved by each user which was shared with other devices of the user. Opportunistic service attack, self-promoting attacks, bad-mouthing, and ballot-stuffing attacks were taken care of in the proposed system. Furthermore, a weighted parameter technique was used. A storage management technique that is important for small devices was also devised. A comparative analysis with EigenTrust and PeerTrust was done. By managing the list at the user end made it difficult to share the experience with those users who are not part of social relations. Therefore, it is difficult to establish more precise trust/reputation. In addition, context-awareness was not given attention.

A Context Awareness architecture for the Internet of Things (CA4IOT) architecture was proposed for task automation by selecting sensors. It reduced the difficulty for users who faced in selecting the appropriate sensor [23]. The proposed architecture consisted of four internal layers: Data, Semantics, and Context Dissemination Layer (DSCDL), Processing and Reasoning Layer (CPRL), Context and Semantic Discovery Layer (CSDL), and Sensor Data Acquisition Layer (SDAL); and two external layers: user layer and sensing layer. The different components and layers worked together for the automation of filtering, fusion and reasoning mechanisms. The results of experiments showed that the proposed architecture had improved the context-aware capabilities of MW and had enabled to establish sensing as a service platform. However, the system did not consider QoS criteria for the trust evaluation.

A framework for context-aware service composition for IoT based smart cities was proposed [24]. The framework was an abstract service model based on wEASEL. The abstract model represented services and specification, signature and conversation represented user tasks. Furthermore, it supported dynamic reasoning. The services were selected against a user’s task and then coordination was made to find various possible combinations to satisfy the user criteria. The framework was evaluated using OWLS-TC4 testbeds by the combination of composite and simple web services. It was identified that no QoS criteria was taken care of in this research.

Apriori Learning and Bayesian Classifier based trust model was proposed in [25]. Two steps were defined for decision-making in which behavioral pattern was identified using a priori learning and Bayesian was used for the decision. In addition, the entity’s experience was presented in the form of a vector for evaluating trust. Various attacks are tackled in the system. However, the research did not focus on context awareness and QoS based trustworthy evaluation. In addition, the interoperability of heterogeneous devices was also not considered in this research.

In [26], a contextual social network model was presented that considered independent social contexts such as role impact factor and preference, and dependent social contexts such as trust and social intimacy degree. A probabilistic approach was presented for social contextual-aware trust interference. An iterative approach was proposed in this research work for contextual trust interfaces to consider cycles along with strong and weak connections. The proposed system provided a solution to overcome data sparsity problems and getting recommendations from unknown participants. The results of the experiments showed that the proposed model yield positive and trustworthy results. However, QoS and interoperability were not able to get the attention of the researchers.

In [27], the context-aware trust-based framework was proposed for the dissemination of information in the vehicular network. The proposed framework called FACT was composed of two modules: (a) admission module and (b) dissemination module. It first checked the authenticity and validity of the message sent. After checking stored trust values, it selected the best path for disseminating the message. The results of the simulation showed that FACT was more effective and scalable than other routing protocols like VADD.

Another research work [28] focusing on privacy preserving data security and mining presented a framework called PPSF (Privacy Preserving and Security Mining Framework). This framework was an open-source library offering various algorithms for Privacy Preserving Utility Mining (PPUM), Privacy Preserving Data Mining (PPDM), and data mining. The framework showed the results after running the algorithm.

In [29], a distributed trust model was proposed that considered the historical data of the nodes. Trust was calculated for a service seeker through a single function. However, interoperability and context awareness were not part of this study. Another trust evaluation model focusing on social IoT and context awareness was presented in [30]. The proposed model maintained quantified trustworthiness of a node in a distributed approach. The attributes such as context importance, energy, computation power and feedback of a service consumer were used as criteria for calculating the trustworthiness. However, it was identified that QoS based evaluation was ignored in the proposed model.

A flexible trust-aware access control system for IoT (TACIoT) was proposed. It provided reliable, secure and end to end mechanism for this purpose [31]. To compute the value of trust about IoT devices, a trust model considered four aspects: (a) reputation, (b) security aspects (c) quality of service and (d) social relationships. The trust model was implemented using fuzzy control systems. The feasibility of the proposed framework was tested by running the software on real scenarios for constrained devices and non-constrained devices; however, the context-awareness gets less attention of the researchers.

Zhou et al. [32] presented a survey that discussed the features of IoT. The research also explored the effects of security and privacy including the threats. Moreover, the state-of-the-art solutions tackling the stated issues were also discussed. Based on this, the research identified the challenges faced by this area and then stated the opportunities.

In [33], a sanitization approach for hiding confidential information by ensuring useful information still available was presented. For balancing four size effects, HCMPSO and particle swarm optimization framework were used. For experimental purposes, NSGA-II and cpGA2DT approaches were used that demonstrated the security of published data in IoT in a way that the information was hidden after sanitization.

A multiobjective algorithm based on NSGA-II algorithm was presented in [34] that had two strategies for hiding sensitive data. More than one solution were provided by the proposed algorithm. The pre-large concept and a Fast SoRting (FSR) strategies were used for improving the iterative process and finding the transaction for deletion. Several Pareto solutions could be found based the proposed algorithm. Moreover, initial weights were not required for assessing the side effects. The experimental validation showed that the algorithm reduced the side effects and proved to be quite efficient.

Frustaci et al. [35] presented the security analysis of three main layers of IoT systems and then highlighted the critical things that are being faced by the researchers in this area. It was identified that trust management and security must be ensured to avoid the exploitation of weaknesses. It was highlighted that the perception layer was the most vulnerable layer because of physical exposure.

## 3. Context Aware QoS Trust Management

The idea is based on providing things as a service in which each thing provides its services. For this, a service-oriented approach i.e., Service Oriented Architecture (SOA) is considered. This scheme is being used in recent research works such as [3,4,8,36].

**Figure 1:**
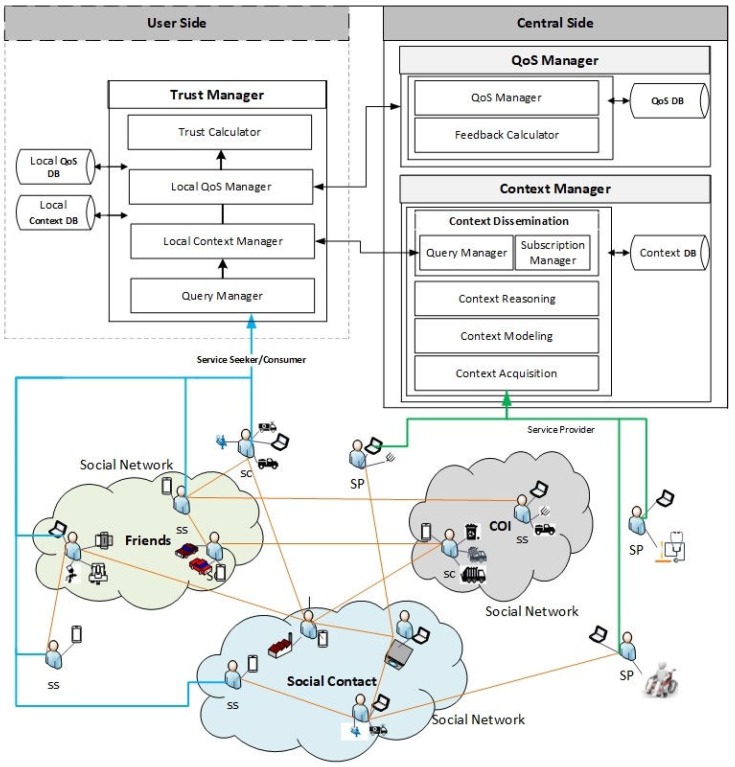
The Context Aware Trustworthy Social Web of Things.

Each service representing a thing has a unique address that in this case is URI (Uniform Resource Identifier). There is both a centralized level and a user level management of trust based on QoS criteria and context awareness that is calculated directly (based on experiences) and indirectly (based on the recommendations from others). For recommendations from others, the idea of Social Web of Things is also exploited. The recommendations get autonomous after the authorization of its contacts. The proposed architecture is shown in Figure 1, which is adapted from [4]. The users and their relations in CATSWoTS are explained in Section 3.1, whereas Section 3.2 describes trust management in the proposed system.

### 3.1. Users and Relations

#### 3.1.1. Types of Users

The term “Service Provider (SP)” is being used for those who are offering their things as services for others. The term “Service Seeker (SS)” is used for those who are searching for a trustworthy service, whereas “Service Consumer (SC)” is being used for those who have used the service and are now in a position to give the feedback or have given the feedback about a service provider. The owner of the devices who wants to sell the services can register things in the form of services on the centralized server. These services can be identified and detected either (i) through social relations (ii) or through a central server. The SC can also be the SP at the same time by offering services to others. The relation between a user and the services is one-to-many. A SP may have many devices or services to offer. A profile is maintained for each device or services at SC level. Similarly, the SC or SS may require many devices or services from others while it has its own devices or services. The terms SP and service are used interchangeably in the paper.

#### 3.1.2. Types of Social Relations

In distributive collaborative filtering, to get recommendations from others, various types of contacts are looked upon, but, in CATSWoTS, three types of relations are considered.

(i) Community of Interest (CoI) (ii) Friendship (iii) Social Contact. A trustor, which is a service seeker in this case, tends to have more confidence over the recommendations of recommenders with whom the trustors share similar interests or have some kind of social relationships as described in [3]. For getting recommendations, a user identifies and allows these relations to be in their contact list. After authentication from the user, recommendations can be obtained from these relations. The authentication and authorization make the system secure as it limits the access only to the known contacts.

CoI refers to the relationship with those who have the knowledge and contribute to the same area. Friendship manifests intimacy among the contacts, whereas social contacts manifest closeness. For this purpose, three lists are maintained for each user in the user’s central device, which are shown in Equations (1)–(3):

(1) $$F=\{f_{1},f_{2},f_{3}\cdots ,f_{n}\},$$

(2) $$C=\{c_{1},c_{2},c_{3}\cdots ,c_{n}\},$$

(3) $$S=\{s_{1},s_{2},s_{3}\cdots ,s_{n}\}.$$

### 3.2. Trust Management

For assisting service seekers, in identifying and selecting context-aware trustworthy services fulfilling the QoS criteria, trust is managed at the centralized and at the user level as well. Besides this, social relations are also considered for evaluating and managing the trust. The QoS manager identifies the services based on criteria defined by an SS. It also maintains the feedback given by a SC. The Context Manager first registers the context of services that are being provided by SP, models it, makes reasoning and then disseminates it, and hence completes the life-cycle of context as described in [5]. The details of the major components of the system are described in the following subsections.

#### 3.2.1. Context Manager

**Figure 2:**
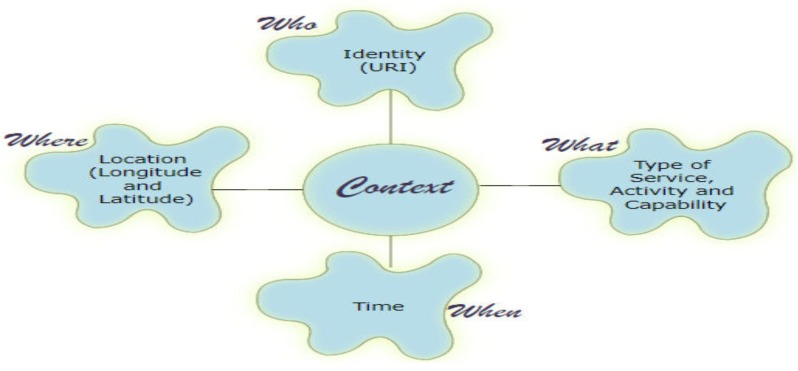
Categories of the context in CATSWoTS.

In CATSWoTS, two context managers at the local level and at the central level are designed. Conceptually, both are doing the same work and are deployed at two different levels. The difference between the two context managers is that the central context manager performs the phases of context acquisition and context modeling. In addition to this, the central context manager also maintains the history of services that were consumed in the past. The context managers consider four categories of the conceptual and the primary category of the operational perspective of the context as described in [5]. Perera et al. identified various categories of context including (i) who; (ii) what; (iii) where; and (iv) when types of context in state of the art [5]. In CATSWoTS, the mentioned types are also considered, the details of which are shown in Figure 2.

Each thing is identified by URI (Uniform Resource Identifier), which covers the aspect of “Who”. For “What”, the service type and the capability (which varies from one service type to the other) are considered as stated in [3]. Location of the things and the location of service seeker, in the form of longitude and latitude, are considered for the “Where”. The time at which service is required manifests the “When”. The aging time can also be used to reflect it.

The first step of context acquisition is completed manually i.e., the service provider itself enters the detail of context information on the centralized server. The details include the registration of the service provider, types of services it provides, URI of each service and activity it performs. Then, the attributes like capability, location and time are also entered in the context manager. For context modeling, markup scheme modeling (e.g., JSON) is used as described in [5]. JSON is preferred over XML because the parsing process of XML takes time. JSON is more readable and can be easily mapped to domain objects.

**Figure 3:**
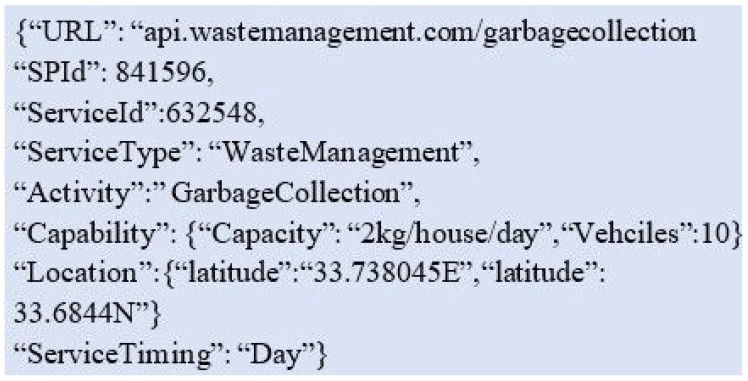
Service modeling at service provider end.

An example of SP service modeling for a smart waste management service provider based on IoT is shown in Figure 3. In this model, all of the information related to W4 is described. For context reasoning, a simple rule-based approach is used as it is the most popular approach in state of the art. The rules are defined based on various parameters.

**Figure 4:**
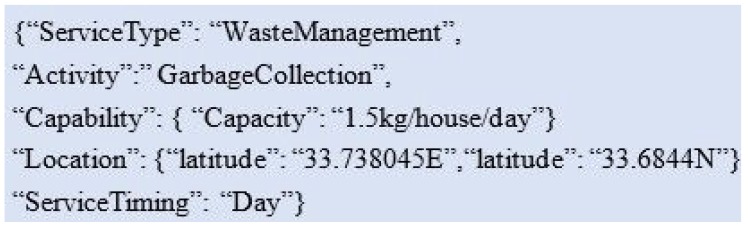
Modeling at service seeker end.

The context of SS is also taken in this pattern which helps in identifying the relevant SP. The example is shown in Figure 4 for an SS who is in search of a trustworthy waste management service provider.

The matching and identification of the services based on the context of the SS is described in Algorithm 1. The SS gives the parameters of δ which is a service category, α depicting service name, Ω manifests capability which may have many variables. As shown in Figure 3, the capability of the smart bin is 2 kg garbage per house per day. The energy/battery level of the sensors embedded in this smart bin might be another capability. Then, the location of a service is identified in the form of longitude λ and latitude ϕ. The importance of the distance between the location of SP and the location at which a service is required cannot be ignored. Therefore, the Haversine formula for distance calculation is considered in the CATSWoTS. The mentioned formula calculates the distance by taking the values in longitude and latitude and measures the distance in kilometers or miles. The “when” of the context can be covered in different aspects like the aging of the device and the time at which a service is required. In this example, the service timing is significant as the waste management company provides service in the morning, afternoon and in the evening.

After getting the input parameters, the local context manager searches its local database for the existence of any service id resembling the context of required service. It then forwards that list of services to local QoS manager for the discovery of services fulfilling the QoS criteria; otherwise, a message is sent to social relations.

In case the required context criteria are neither found in local context manager of SS nor its social circle, then the request goes to the central level. The central context manager, which maintains a huge database of service providers, repeats the process, and identifies the potential services. The process for services identification based on context is described in Algorithm 1.

**Algorithm 1** Services identification based on context
Get the values of
δ and αΩ = Ω1, Ω2, ..., ΩnΦ = Φ1, Φ2 and λ = λ1, λ2
i←0

**for** each service ’S’ **do**
 **if** S. δ == δ and S. α == α **then**  **for** (j←1; j<N; j++) **do**   **if** (Ω*j*!=S. Ω*j*) *break* **then**    **else** *continue*   **end if**   make a list  **end for**  **if** (τ ==S. τ) **then**   Δ λ←λ2− λ1   Δ Φ←Φ2− Φ1   *a*←(sin(ΔΦ/2))*(sin(ΔΦ/2))+cos(Φ1)*cos(Φ2)* (sin(Δλ/2))*(sin(Δλ/2))   c←2*atan2(sqrt(a),sqrt(1−a))   d←R*c   sURL[i]←S.url   i++   sort(sURL[i])  **end if** **end if**
**end for**



The context manager generates a list of those services that have a similar context, which is required by an SS. This list is sorted by the distance between an SS (where a service is required) and an SP (the location of the service) in descending form i.e., services with minimum distance will be on the top. This list is then forwarded to QoS manager for the evaluation of QoS criteria on the basis of required criteria of an SS. The list may be either generated by the local context manager of an SS or the local context manager of the contacts in a social network or the central server.

The lists, which are received from recommenders including social relations and central level, are then consolidated at the local level.

#### 3.2.2. Social Relations

As mentioned before, if a local context manager does not find any service resembling the required context criteria in its local database, then a broadcast message is sent to the social circle for the recommendation of trust-worthy context-aware SPs. Each contact of an SS receives the query and then a local context manager of each contact repeats the process that is described for the local context manager of an SS. If the required context criteria are met, then the local QoS manager of that contact does the further processing.

#### 3.2.3. QoS Manager

Two categories of QoS constraints (positive and negative) are found in the state of the art as described in [37]. Examples of positive constraints are availability, reliability, performance, and throughput. The high value of a positive constraint means high quality of service, whereas the high value of a negative constraint means the low quality of service. Latency and cost are the two examples of negative constraints. There can be different positive and negative constraints that can help with evaluating the quality services. Reputation is also another criterion that is found in the literature, which helps in evaluating trust. The QoS constraints that are considered in the CATSWoTS are shown in Table 1.

QoS managers are also part of both user level and central level. The algorithm for QoS based identification is shown in Algorithm 2

**Algorithm 2** Services identification based on QoS
Get the weightage of quality parameters
**for** each service ‘SL’ in the list **do**
 **for** (k←1; k<N; k++) **do**  **if** (qk!=SL.qk) **then**   break  **else if** continue **then**   **end if** **end for** **if** required service is found at local base **then**  Show the previous experience **else if** required service is recommended **then**  calculate R **end if** **if** more than one service fulfills the criteria. **then**  merge the list and sort it in descending order **end if**
**end for**



The QoS managers perform two major tasks:

1. Evaluation of services based on QoS criteria,
2. Feedback calculation and updating the record.

As per SS requirements, services are identified from the shortlisted services fulfilling QoS constraints. The services that are found in the database of an SS means that the SS has already directly experienced the quality of such services and the trust level of such services is already maintained.

If the required service is not found in the database of an SS, then a broadcast request is sent to a social circle for the recommendations. If the services are found in different social relations, then lists of recommendations are returned to the SS from its social network. These lists are sent in sorted form displaying the services fulfilling the required criteria of Context-Aware QoS. After receiving the list, the local QoS manager at SS end considers this recommendation by applying a mathematical model shown in Equation (4).

(4) $$R=(\sum_{i=1}^{N}q_{1}+\sum_{i=1}^{N}q_{2}+\sum_{i=1}^{N}q_{n})/N.$$

The combined list of recommended services is then shown to SS. The list is sorted in descending order in which services with minimum distance is shown on the top.

After consuming a service, the SS that is now an SC gives feedback about the quality of the service. A profile is maintained for the consumed service in the local database of SC and the feedback is also sent to the central server, hence it can be presented as an indirect trust to another SS.

Trust is calculated through the feedback given against each QoS criterion and then Fij, the aggregated value for each QoS constraint, is updated with Equation (5):

(5) $$F_{ij}=\left(\left(\omega_{i}X_{ij}\right)+F_{ij}\right)/t_{ij},$$

where the weight of each criterion allocated by the service seeker is denoted by ’ω’ whereas the feedback assigned by the service seeker, for constraint ‘*i*’ satisfied by the device ’*j*’, is denoted by $X_{ij}$ is and for how many times the device ’*j*’ has been evaluated for the constraint ’*i*’ is symbolized by $t_{ij}$. The total value of trust ’$T_{j}$’ is calculated for service provider *j* with Equation (6):

(6) $$T_{j}=\sum F_{ij}.$$

**Figure 5:**
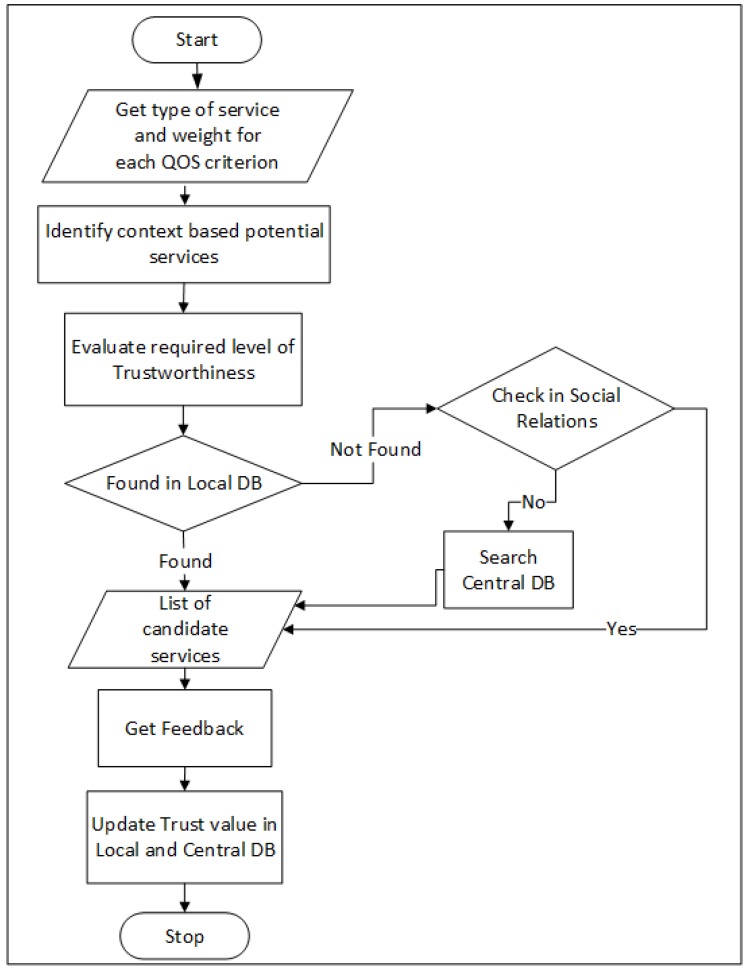
Flow chart summarizing the whole process.

The whole process of identifying potential services, then getting feedback and updating it is summarized in the form of flowchart shown in Figure 5.

## 4. Experimental Validation and Results Discussion

This section presents the efficacy of our proposed system. To evaluate the performance and prove the effectiveness of CATSWoTS, the validation was done in a specifically designed environment. For experimental setup, the following roles were developed:

1. A central server where a context manager and a QoS manager resides. An SP may register the services there.
2. The nodes that may play the role of service providers and service consumers as well. These nodes are also connected through a peer to peer network. Each node has two local managers for context, QoS and trust management. It has its own local database on which the profiles of consumed services are maintained.

The trust-based invocation is generated based on real dataset [38]. The feedback about the QoS of 2507 services were used in this experiment. At the server level, the profile of 2507 services resides, whereas each node contains a dataset of 50–100 profile of each consumed service. The nodes can communicate with each other and with the central server as well. The available dataset evaluated the services with respect to QoS constraints only; however, the context related attributes are added by considering the general criteria so that it can be applied to any kind of service for IoT and to evaluate the effectiveness of CATSWoTS. Therefore, the following attributes have been added to accommodate the context awareness specifically related to IoT services.

1. Capacity: A set of attributes defining the capacity of any service may include battery power, processing speed, memory, and another service-related capability. For example, Alice wants the garbage collection services of IoT based waste management company. In this case, the attributes may include smart bin’s capacity to contain garbage in kilograms, battery, processing power and memory of sensors and actuators that constantly monitor the level of garbage in the bin and then intimate the SP when it needs to empty the bin. However, to keep the attribute simple and generic for testing purposes, three levels of capacity are defined which are (i) low, (ii) medium (iii) and high.
2. Location: Location, where the services are provided, is described in terms of longitude and latitude.
3. Time: the timings of services are described as (i) morning, (ii) noon (iii) evening and (iv) 24 h like the waste management company may provide the services in the morning but Alice wants the services in the noon then Alice is in the search of an SP which offers the services in noon.

A social IoT environment with *n* = 30 nodes is created. These nodes form a social circle with each other. Thirty nodes are further divided into three subgroups to send multiple requests from different social groups. In Equations (1)–(3), three lists are being maintained. Some nodes are directly connected with each other, whereas others are indirectly connected through their circle with each other. As described previously, each node is assumed to consume 50–100 services and hence maintains the feedback of these services, which is considered as direct trust. Any service seeking node requests the recommendation of a particular service and, upon consumption of service, direct trust assessment will be updated, whereas indirect assessment from where the recommendation comes will be updated using Equations (4) and (5). Trust value ranges from 0 to 1 where 0 means not satisfied, 0.5 means satisfied and 1 means highly satisfied. Initially, $T_{j}$ in (6) is set to 0.5. Gradually, trust is built gradually when nodes interact with each other by seeking and consuming services after getting feedback.

The proposed concept is evaluated based on its core ideas which are context awareness and QoS constraints-based trust management. It is done by experimenting at SS and SP nodes and then identifying the potential services based on the prescribed criteria. Since the dataset has different categories of services such as commerce, business, management, Google, and Amazon. However, the experiments are conducted on the first three categories by varying the input parameters given at a service seeker’s end. The details of these experiments are described in the following subsections.

### 4.1. Effect of Context Aware Attributes

**Figure 6:**
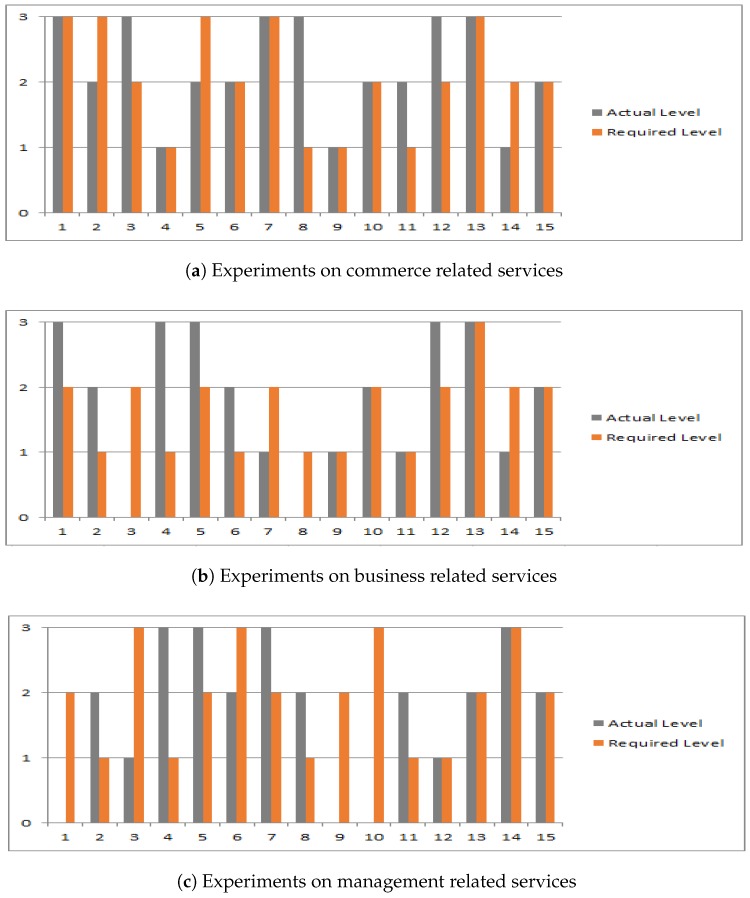
Context aware evaluation.

Fifteen queries were executed for each category (commerce, business, and management) to check the effectiveness of the proposed algorithms. The analysis of the summary of multiple requests with different values of context attributes is shown in Figure 6. The *x*-axis shows the number of queries along with the request and response. The *y*-axis shows the context level of a service where “required” level manifests the level desired by a service seeker and maximum “actual” level, service is providing among the identified shortlisted services in each request. Level 1 of the context on the *y*-axis shows that fulfilling only one criterion of context parameters like type is the minimum threshold, whereas level 2 means that two parameters (like type and capability) out of three are met. Level 3 means all three parameters of context are to be met. In the randomly generated requests for each category, the service seekers were in search of services of a particular type with various values of other two criteria. The thirty nodes were distributed in three groups connected through social networks. The system recommended the few services after shortlisting the desired services among a pool of potential services against each request. The parts of figures manifest the results obtained in multiple experiments for three categories. Figure 6a shows the the requests and the response received with respect to context levels for commerce related queries. These services were required in different timings like morning, noon and evening. Hence, the requests contained different combinations of context parameters. For example, an SS (who was a big manufacturing company) required commerce services in the morning and asked for the recommendation of an SP with high capacity. The CATSWoTS shortlisted top service providers which fulfilled the criteria level required by the SS. i.e., the shortlisted service providers were offering the commerce services and these SP could have highly skilled employees. It is evident from Figure 6a that the results of four queries recommended at least one service whose criteria were above the required level. Eight queries recommended at least one service fulfilling the exact criteria, whereas three queries were not able to discover any required service.

The distance between SP and SS was measured with the help of longitude and latitude of both SS and SP. An SS can mention the distance limit for a service provider. In this request, various values of distance were specified by the service seeker, hence these shortlisted services were providing services within the required kilometers. These shortlisted services were then forwarded to the next phase for matching the QoS attributes. Figure 6b,c show the results of fifteen sets of queries for business and management categories.

It was observed that the context-aware attributes proved to be quite effective in identifying the context of trust for which a service is required. This is the reason that the required service providers were shortlisted based on context parameters. It is evident that the system recommended the the service providers to be equal to or above the required level. It is established that context awareness plays a significant role in identifying trustworthy IoT services [5]; however, it is noteworthy to mention that all the context based parameters (proposed in CATSWoTS), especially service timings, are not considered in state of the art for WoT. Without considering the aspect of context, trustworthy IoT services cannot be recommended. This is the reason, in CATSWoTS, that the concept specifically dealing with WoT has been considered hence enhances the novelty of the system.

### 4.2. Effect of QoS Constraints

The results presented in this section are based on the experiments conducted in Section 4.1. The lists generated based on various values of context parameters were then used to evaluate the shortlisted service providers based on QoS attributes.

**Figure 7:**
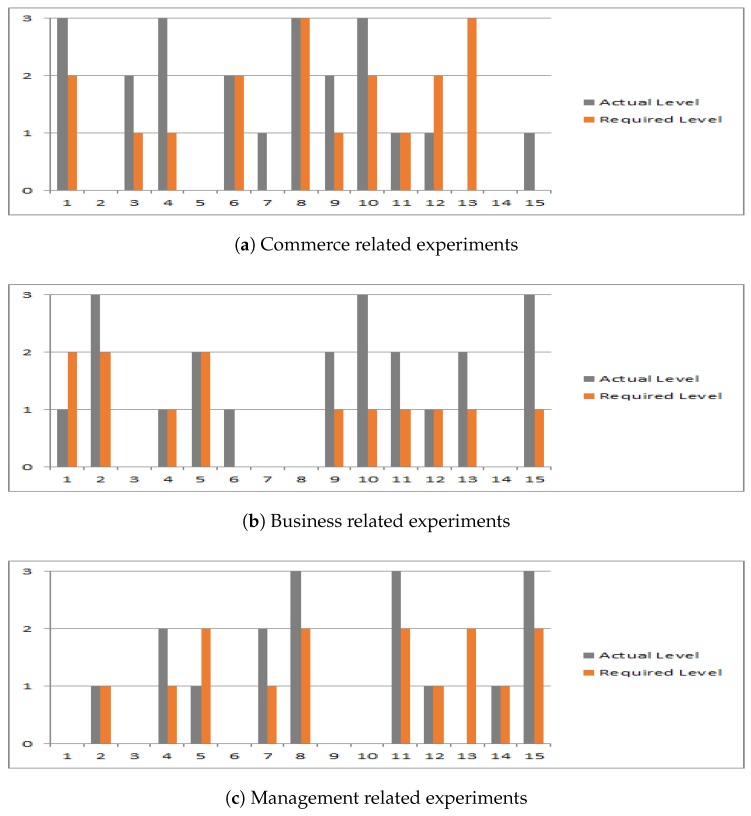
QoS evaluation.

The *x*-axis shows the number of requests generated. The *y*-axis shows the QoS level of a service where “required” level manifests the level desired by a service seeker and maximum “received” level, a service provided from among the identified services in each request. The real dataset used in the evaluation acts as a ground truth. For testing purposes, we have considered three attributes (reliability, availability, and latency). A service seeking node entered the weight of each criterion based on the requirements. For example, the SS wanted the services of a commerce company always available for them. Therefore, the SS gave more weight to availability than reliability. The shortlisted services are then evaluated based on Equation (5) and trust level is calculated for each service based on Equation (6). The CATSWoTS gave the sorted list of service providers that were close to the proximity of the SS. The same process of evaluating all the shortlisted service providers for different values of the QoS attributes was completed in this phase. It was observed that not all the shortlisted services fulfilled the criteria required by the SS. Figure 7a shows the final results for the commerce category. It is significant to note that level 1 shows that the identified service fulfills only one QoS criterion required by the SS, level 2 manifests that the identified service fulfills two QoS criteria, whereas level 3 symbolizes that the identified service fulfills all three criteria of QoS. It is evident that seven queries recommended one service at least, which was above the criteria. Four queries presented at least one service that fulfilled the exact criteria, whereas four queries were not able to identify any required service. Similarly, Figure 6b,c show the queries’ results for the business and management categories, respectively.

Though the experiments are carried out on three attributes of QoS, more attributes like throughput and response time can be added in CATSWoTS. In short, adding more attributes in CATSWoTS does not require any additional effort, but service providers can be evaluated on more criteria. The evaluation of service providers in the specific context of trust along with the criteria of QoS is a contribution of the CATSWoTS in finding a trustworthy Web of Things.

### 4.3. Effect of Social Relations

**Figure 8:**
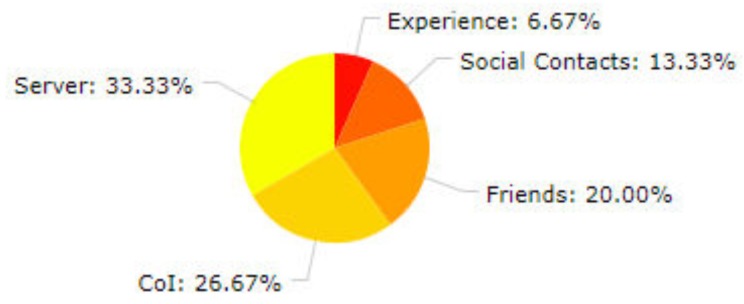
Analysis of recommendations.

The CATSWoTS exploits the concept of Social Web of Things for identifying and recommending trustworthy services. The flow of the getting recommendations is shown in Algorithm 3. The effect of incorporating the social aspect was also evaluated during the various experiments mentioned in Section 4.1. In case of a request for identifying and recommending services of a waste management company, five out of eight shortlisted services were recommended by social relations. During experiments, it was evident that the social relations, proposed in CATSWoTS, proved to be beneficial. In case a request was generated by SS, if no experience of the desired service was there, then the recommendations were made by social relations. Figure 8 shows the percentage of recommendations from (i) social relations (ii) and the server that appears in the response to various queries during experimental evaluation.

**Algorithm 3** Interaction between the nodes
Request for service recommendation**for** ∀ services in local database **do** search(required service ‘S’) **if** found==true **then**  insert(potentialServiceList) **else if** *break* **then**  **end if**
**end for**
**for** ∀ contacts in social circle **do** sendQuery(required service ‘S’) **for** ∀ contacts who return a list of potential service list **do**  insert(potentialServiceList) **end for**
**end for**
sendQueryToCentralSide(required service ‘S’)**if** CentralSide returns a list **then** insert(potentialServiceList)
**end if**
sort(potentialServiceList, criteria)


### 4.4. Comparison with State of the Art

The novelty of the recommender system is compared with the related state of the art. It is observable from Table 2 that, to the best of our knowledge, there was a lack of such a system in the state of the art which covers context awareness of trust while considering the quality of service for recommending WoT based trustworthy service providers. It was found that the attention of many researchers was privacy and security [39], whereas other qualities of service attributes like availability and reliability were not focused on much. In addition, an important aspect of context dependency of trust was also less considered in these types of research. Similarly, the social aspect has been the focus of the researchers to less extent in research where the aspects of context and QoS were considered. It is interesting to note that [31] considered the social dimension for IoT in the sense that it formed the bubbles of things for friends and family; then, these things were evaluated for access control. However, in the case of absence of any required thing belonging to friends and family, the recommendations from social networks were not taken into consideration. Keeping more focus on the era of social networking while identifying and recommending trustworthy WoT among billions of service providers is the need of the time. Consequently, the CATSWoTS focuses not only on social aspects but also on all the aspects mentioned and fulfills these gaps present in the current state of the art.

## 5. Conclusions and Future Work

In this paper, context-aware QoS based trust management for SOA based WoT was designed and analyzed. An SOA approach resolves the interoperability issues of IoT. Trust management considers context awareness and QoS constraints for evaluating the trustworthiness. Context awareness helps in the identification of distance-based services having the required capacity and time hence suitable in a specific context. By considering QoS constraints, CATSWoTS ensures the identification of quality services. For this, a centralized and distributed collaborative filtering technique was used to get trust recommendations from the nodes sharing similar social relations. Trust is managed and evaluated at (i) a node (ii) and a central level as well to maximize the efficiency. During experimental validation, it is observed that the system discovers and ranks the services out of a large dataset based on the user defined criteria of context and QoS. The history of the each consumed service is maintained by taking the feedback from a service consumer that is also aggregated and incorporated, which keeps the performance and trustworthiness of a thing up to date; hence, any service seeker gets the updated trustworthiness of a thing. In short, the use of social aspects in WoT for the recommendation and evaluation of trustworthy things based on the criteria of context awareness and QoS is a contribution of this research. In the future, more parameters for context awareness can be identified. The system can be made autonomous and intelligent for the identification and evaluation of trustworthy services.

## Figures and Tables

**Table 1:** Quality of service constraints definitions.

QoS Constraints	Definition
Reliability	Reliability degree for service
Availability	Existence and availability of the service.
Latency	The interval between stimulation and response
Reputation	Feedback score is given by service consumers to a web service

**Table 2:** Comparison with related systems.

Prev Work	Authenticity, Privacy or Security	Availability and Reliability	Social Recommendations	Context Aware
[16]	Yes	No	No	No
[17]	No	No	To some extent	No
[18]	Yes	To some extent	No	No
[20]	Yes	No	No	No
[22]	No	Yes	No	only capability, type
[23]	No	No	No	Yes
[24]	No	No	No	Yes
[26]	No	No	Yes	Yes
[27]	Yes	No	No	Yes
[31]	Yes	Yes	Yes	No
CATSWoTS	Yes	Yes	Yes	Yes

## References

[1] Chen R., Bao F., Guo J. (2016). Trust-based service management for social internet of things systems. IEEE Trans. Dependable Secur. Comput..

[2] Kamilaris A. (2013). Enabling Smart Homes Using Web Technologies. Ph.D. Thesis.

[3] Chen R., Guo J., Bao F. (2016). Trust management for soa-based iot and its application to service composition. IEEE Trans. Serv. Comput..

[4] Varga P., Blomstedt F., Ferreira L., Eliasson J., Johansson M., Delsing J., de Soria I. (2017). Making system of systems interoperable—The core components of the arrowhead framework. J. Netw. Comput. Appl..

[5] Perera C., Zaslavsky A., Christen P., Georgakopoulos D. (2014). Context aware computing for the internet of things: A survey. IEEE Commun. Surv. Tutor..

[6] Atzori L., Iera A., Morabito G. (2010). The internet of things: A survey. Comput. Netw..

[7] Atzori L., Iera A., Morabito G., Nitti M. (2012). The social internet of things (siot)–when social networks meet the internet of things: Concept, architecture and network characterization. Comput. Netw..

[8] Usman M., Zhang X., Chiroma H., Abubakar A., Gital A. (2014). A framework for realizing universal standardization for internet of things. J. Ind. Intell. Inf..

[9] Guinard D., Trifa V., Mattern F., Wilde E. (2011). From the internet of things to the web of things: Resource-oriented architecture and best practices. Architecting the Internet of Things.

[10] Atzori L., Iera A., Morabito G. (2014). “From” smart objects to social objects: The next evolutionary step of the internet of things. IEEE Commun. Mag..

[11] Andročec D., Novak M., Oreški D. (2018). Using semantic web for internet of things interoperability: A systematic review. Int. J. Semant. Web Inf. Syst. (IJSWIS).

[12] Aftab S., Afzal H., Khalid A. (2015). An approach for secure semantic data integration at data as a service (daas) layer. Int. J. Inf. Educ. Technol..

[13] Javaid S., Majeed A., Afzal H. A reputation management system for efficient selection of disaster management team. Proceedings of the 2013 15th International Conference on Advanced Communications Technology (ICACT).

[14] Guo J., Chen R., Tsai J. (2017). A survey of trust computation models for service management in internet of things systems. Comput. Commun..

[15] Li C., Palanisamy B. (2019). Privacy in internet of things: from principles to technologies. IEEE Internet Things J..

[16] Sato H., Kanai A., Tanimoto S., Kobayashi T. Establishing trust in the emerging era of iot. Proceedings of the 2016 IEEE Symposium on Service-Oriented System Engineering (SOSE).

[17] Bao F., Chen I.-R. Dynamic trust management for internet of things applications. Proceedings of the 2012 International Workshop on Self-Aware Internet of Things.

[18] Ning H., Liu H. (2012). Cyber-physical-social based security architecture for future internet of things. Adv. Internet Things.

[19] Roman R., Zhou J., Lopez J. (2013). On the features and challenges of security and privacy in distributed internet of things. Comput. Netw..

[20] Kothmayr T., Schmitt C., Hu W., Brünig M., Carle G. (2013). Dtls based security and two-way authentication for the internet of things. Ad Hoc Netw..

[21] Kumar S., Anouncia S. (2018). Qos-based concurrent user-service grouping for web service recommendation. Autom. Control Comput. Sci..

[22] Saied Y., Olivereau A., Zeghlache D., Laurent M. (2013). Trust management system design for the internet of things: A context-aware and multi-service approach. Comput. Secur..

[23] Perera C., Zaslavsky A., Christen P., Georgakopoulos D. Ca4iot: Context awareness for internet of things. Proceedings of the 2012 IEEE International Conference on Green Computing and Communications (GreenCom).

[24] Urbieta A., González-Beltrán A., Mokhtar S., Hossain M., Capra L. (2017). Adaptive and context-aware service composition for iot-based smart cities. Future Gener. Comput. Syst..

[25] D’Angelo G., Rampone S., Palmieri F. (2017). Developing a trust model for pervasive computing based on apriori association rules learning and bayesian classification. Soft Comput..

[26] Wang Y., Li L., Liu G. (2015). Social context-aware trust inference for trust enhancement in social network based recommendations on service providers. World Wide Web.

[27] Rostamzadeh K., Nicanfar H., Torabi N., Gopalakrishnan S., Leung V. (2015). A context-aware trust-based information dissemination framework for vehicular networks. IEEE Internet Things J..

[28] Lin J.-W., Fournier-Viger P., Wu L., Gan W., Djenouri Y., Zhang J. Ppsf: An open-source privacy-preserving and security mining framework. Proceedings of the 2018 IEEE International Conference on Data Mining Workshops (ICDMW).

[29] De Meo P., Messina F., Postorino M., Rosaci D., Sarné G. A reputation framework to share resources into iot-based environments. Proceedings of the 2017 IEEE 14th International Conference on Networking, Sensing and Control (ICNSC).

[30] Rafey S., Abdel-Hamid A., El-Nasr M. Cbstm-iot: Context-based social trust model for the internet of things. Proceedings of the 2016 International Conference on Selected Topics in Mobile & Wireless Networking (MoWNeT).

[31] Bernabe J., Ramos J., Gomez A. (2016). Taciot: Multidimensional trust-aware access control system for the internet of things. Soft Comput..

[32] Zhou W., Jia Y., Peng A., Zhang Y., Liu P. (2018). The effect of iot new features on security and privacy: New threats, existing solutions, and challenges yet to be solved. IEEE Internet Things J..

[33] Lin J.-W., Wu J.-T., Fournier-Viger P., Djenouri Y., Chen C.-H., Zhang Y. (2019). A sanitization approach to secure shared data in an iot environment. IEEE Access.

[34] Lin J.-W., Zhang Y., Zhang B., Fournier-Viger P., Djenouri Y. (2019). Hiding sensitive itemsets with multiple objective optimization. Soft Computing.

[35] Frustaci M., Pace P., Aloi G., Fortino G. (2017). Evaluating critical security issues of the iot world: Present and future challenges. IEEE Internet Things J..

[36] Rangarajan S. (2018). Qos-Based Web Service Discovery and Selection Using Machine Learning. arXiv.

[37] Modi K., Garg S. (2015). Dynamic web services composition using optimization approach. Int. J. Comput. Sci. Commun..

[38] Al-Masri E., Mahmoud Q.H. The Qws Dataset. http://www.uoguelph.ca/~qmahmoud/qws/dataset/.

[39] Javaid S., Afzal H., Babar M., Arif F., Tan Z., Jan M. (2019). ARCA-IoT: An Attack-Resilient Cloud-Assisted IoT System. IEEE Access..

